# Foliar Nutrition Strategies for Enhancing Phenolic and Amino Acid Content in Olive Leaves

**DOI:** 10.3390/plants13243514

**Published:** 2024-12-16

**Authors:** Marija Polić Pasković, Mirjana Herak Ćustić, Igor Lukić, Šime Marcelić, Paula Žurga, Nikolina Vidović, Nikola Major, Smiljana Goreta Ban, Marija Pecina, Josip Ražov, Matevž Likar, Paula Pongrac, Igor Pasković

**Affiliations:** 1 Department of Agriculture and Nutrition, Institute of Agriculture and Tourism, Karla Huguesa 8, 52440 Poreč, Croatia; igor@iptpo.hr (I.L.); nikola@iptpo.hr (N.M.); smilja@iptpo.hr (S.G.B.); 2 Faculty of Agriculture, University of Zagreb, Svetošimunska 25, 10000 Zagreb, Croatia; mcustic@agr.hr (M.H.Ć.); mpecina@agr.hr (M.P.); 3 Department for Ecology, Agronomy and Aquaculture, University of Zadar, Trg Kneza Višeslava 9, 23000 Zadar, Croatia; simemarcelic@unizd.hr; 4 Teaching Institute of Public Health of Primorsko-Goranska County, Krešimirova 52a, 51000 Rijeka, Croatia; paula.zurga@zzjzpgz.hr; 5 Faculty of Biotechnology and Drug Development, University of Rijeka, Radmile Matejčić 2, 51 000 Rijeka, Croatia; nikolina.vidovic@uniri.hr; 6 Independent Researcher, 23000 Zadar, Croatia; josip.razov@ferotrap.hr; 7 Department of Biology, Biotechnical Faculty, University of Ljubljana, Jamnikarjeva 111, 1000 Ljubljana, Slovenia; matevz.likar@bf.uni-lj.si (M.L.); paula.pongrac@bf.uni-lj.si (P.P.); 8 Department of Low and Medium Energy Physics, Jožef Stefan Institute, Jamova 39, 1000 Ljubljana, Slovenia

**Keywords:** *Olea europaea* L., Leccino cv., verbascoside, oleuropein, oleacein, biostimulants, selenium, silicon

## Abstract

Studies on selenium (Se) and silicon (Si) foliar biostimulation of different plants have been shown to affect concentrations of phenolic compounds. However, their effects on olive (*Olea europaea* L.) primary and secondary metabolites have not been fully investigated. Therefore, the effects of foliar sprayed Si and Se and their combination on the concentration of phenols, selected metabolites involved in the phenol biosynthesis, and mineral elements concentrations were determined in olive leaves of the field-grown cultivar Leccino. During the summer period, leaves were foliar sprayed three times, after which were sampled 30 days after the corresponding application. In general, foliar treatment of Si or Se increased the concentrations of several predominant phenolic compounds, such as oleuropein, oleacein, and specific flavonoids. The effects were especially pronounced after the third application in the harvest time sampling time. Amino acids and other phenol precursors were also significantly affected. The effects were phenol-specific and depended on the treatment, sampling time, and treatment × sampling time interaction. The response of verbascoside to the applied treatments appeared to be closely linked to corresponding changes in its amino acid precursors, such as tyrosine, while its connection with tryptophan and IAA has to be cautiously considered. In contrast, for other phenolic compounds like secoiridoids, a clear interdependence with their precursors was not identified, likely due to the more complex nature of their biosynthesis. The effects on the concentrations of elements other than Se and Si were milder.

## 1. Introduction

Olive (*Olea europaea* L.) is one of the leading fruit species in the Mediterranean, inseparably linked to agriculture but also to the culture and religion of Western civilization. Along with olive oil and table olives, the focus of recent scientific research has been on olive leaves, which, in addition to their important physiological role for the plant itself, are increasingly used as a raw material in various branches of industry. A review of the available literature revealed the use of olive leaves in the production of livestock feed, fertilizers and compost, new materials or energy, and pharmaceutical and food products [1]. Olives are harvested when the fruit is technologically ripe, which in Croatian agroecological conditions is mainly during October, with olive leaves as a by-product with up to 10% of total weight [2]. Because of their antioxidant properties, olive leaves are today used in the production of various functional foods, food supplements, phytochemicals, and nutraceuticals, inseparably connecting science and industry.

Phenolic compounds are among the most important secondary metabolites with biological activity in leaves. Olive is known for its high proportion of phenolic compounds in organic matter and is characterized by the presence of secoiridoids, a group of phenolic compounds specific to the Oleaceae family, among which oleuropein stands out as the most abundant [3]. Precisely because of its specific phenolic composition, which, in addition to oleuropein, includes other oleosides such as oleacein, flavone-7-glucosides of luteolin and apigenin, verbascoside, etc., olive leaves are used in traditional herbal medicine in the Mediterranean. The biosynthesis of phenols in plants is most commonly linked with the shikimate pathway and phenylpropanoid metabolism, which are responsible for the synthesis of aromatic amino acids (AAA) [4]. Aromatic amino acids, such as phenylalanine (Phe) and tyrosine (Tyr), play a significant direct or indirect role in the biosynthesis of all the above-mentioned phenolic compounds found in olive [5]. Another AAA, tryptophan (Trp), serves more as an indirect precursor to phenols. It is utilized for the biosynthesis of indole-3-acetic acid (IAA), a hormone crucial for olive alternative bearing cycles and response to stress [6,7]. The concentration of IAA is also associated with the biosynthesis of phenols [7].

Foliar absorption mechanisms are complex and not yet fully understood. Nutrients applied to leaves can penetrate through the cuticle, stomata, trichomes, and other epidermal structures. The cuticle and cell wall are negatively charged, enabling them to act as a cation exchange membrane [8]. Schönherr [9] stated that in foliar nutrition, salt anions and cations enter the leaf in equivalent amounts to maintain electrical neutrality. However, Bahamonde et al. [8] demonstrated that ion exchange between the leaf and nutrient solution is more intricate. They proposed that certain ions in the applied solution can alter the leaf’s ion balance and the physico-chemical properties of the remaining solution on the leaf surface, thereby affecting the uptake of other ions. Several factors influence nutrient uptake of foliarly applied elements on olive leaves, including leaf characteristics (e.g., surface structure, permeability, and wettability), nutrient formulation (e.g., the type of cations and anions, along with the hygroscopic properties of the salts), and environmental conditions such as temperature and humidity [8,10].

Du Jardin [11] defined a plant biostimulant as any substance or microorganism that, upon application, improves plant nutritional efficiency, tolerance to abiotic stress, and/or quality properties, regardless of its nutrient content. Elements silicon (Si) and selenium (Se) in inorganic form have also been included in one of the seven groups of biostimulants, according to Du Jardin [11]. Although these elements are not considered essential for the plant life cycle, they have been shown to positively affect various production properties [12].

Kaur et al. [13] stated that the lack of Si in plants has a negative effect on growth, development, and reproduction, classifying it as a “quasi-essential” element. In some plants, Si improves structural strength and plays an active role in many physiological processes. When plants are exposed to abiotic and biotic stress, Si increases plant resistance and reduces negative effects [14]. This is the result of two main mechanisms: physical and mechanical protection by SiO_2_ deposition, and biochemical responses that trigger metabolic changes. Among other effects, the role of exogenous Si in plant response to stressors is by upregulating the genes involved in secondary metabolism processes leading to the production of phenolic compounds, which has been systematically reviewed by Ahanger et al. [15]. Silicon can be foliar applied in the form of silicates, (stabilized) silicic acid, and certain other Si-containing compounds [14]. The forms of potassium (K_2_SiO_3_) or sodium silicate (Na_2_SiO_3_) are most often used in foliar fertilizers [12].

Selenium is considered an element beneficial for plants as it has been shown to enhance antioxidant metabolism as well as concentrations of secondary metabolites in plant leaves [16]. Selenium and sulfur (S) share similar chemical characteristics, and both occur in the same oxidation states, such as −2 for selenide (Se^2−^), +4 for selenite (SeO_3_^2−^), and +6 for selenate (SeO_4_^2−^). At the same time, selenate is a chemical analog of sulfate, and they compete for the same transporters during root uptake, so the uptake of selenate can be reduced under the influence of high sulfate application [17]. Several studies reported an increase in the content of phenolic compounds in tissues of various plants after fertilization with Se, although the response was not always proportional to the dose [18,19,20,21] Supplementation with Se has been shown to increase the expression of genes and the activity of enzymes that catalyze the biosynthesis of various groups of phenols, including phenylalanine ammonia-lyase (PAL), a key enzyme in the phenylpropanoid pathway [18,21]. The biofortification of foods and related products with Se has an advantage compared to direct supplementation of Se in the diet because inorganic Se forms absorbed by the plant are converted into organic ones of greater bioavailability.

So far, most studies with foliar application of Se or Si have been conducted on herbaceous crops, while only a few on fruit trees [22,23,24]. Although foliar fertilization results in the most efficient absorption of nutrients, foliar application of beneficial elements such as Si and Se on olive and its products has not yet been thoroughly studied, and only a few studies have addressed its effects. Martos-García et al. [25] found that Si foliar application promotes growth in olive plants, while Hassan et al. [26] showed that it can improve its physiological and biochemical characteristics. Franić and Pasković et al. [27] demonstrated that Si foliar application positively influenced the yield of cv. Leccino olives while maintaining extra virgin olive oil (EVVO) quality parameters without any adverse effects. D’Amato et al. [28] observed that foliar Se supplementation mitigates water deficiency stress and influences olive tree production, as well as improves olive oil stability against oxidation by increasing the content of antioxidant phenolic compounds.

Besides the positive effects of separate foliar treatments with Se and Si, numerous studies have demonstrated that the combined supplementation with these two elements induces phytohormonal and antioxidant stress signaling mechanisms in a variety of plants, as reviewed by Kapoor et al. [29]. The authors noted that the interplay of Se and Si exhibit similar induction pathways in many plants, though their effectiveness varies among different species. Among other effects, it was shown that the foliar application of Se/SiO_2_ nanoparticles results in the upregulation of phenol production in strawberries under stress conditions [30].

To our knowledge, none of the previous reports addressed the effects of foliar application of Se and Si on temporal variation of important secondary metabolites in olive leaves. Based on the current knowledge of their possible impacts on plant metabolism, experiments on olive trees (cv. Leccino) were conducted to test three main hypotheses:

(i) Foliar application of Se, Si, and their combination enhances the synthesis and increases the concentrations of phenolic compounds, especially verbascoside, oleuropein, oleacein, luteolin, and apigenin glucosides in olive leaves;
(ii) Such increase can be associated with the changes in the levels of specific AAA phenol precursors, such as Phe and Tyr, as well as other precursors, intermediates, and other compounds involved in the corresponding biosynthetic pathways, such as Trp, shikimic, and quinic acids, and IAA;
(iii) Foliar application of Se, Si, and their combination will affect the elemental composition of leaves beyond increasing the Se and/or Si concentration.

## 2. Results

### 2.1. Concentrations of Phenolic Compounds in Olive Leaves

The applied foliar treatments (T) did not result in significant differences in total phenolic compounds and antioxidant activity in olive leaves, but there were significant effects of sampling times (ST) (I-August; II-September; III-October) (Table 1). The highest total phenolic compound concentration and FRAP values were found at ST-III compared to ST-I and ST-II, respectively. The interaction between T × ST for DPPH and for the sum of HPLC phenols was significant, so multiple comparisons analysis was performed by RM ANOVA, revealing a variety of statistical differences between means (Table 1). When compared to Control × ST-I, Control × ST-II, and Control × ST-III, a higher sum of HPLC phenols values was determined for (Se+Si) × ST-I, Si × ST-II, and (Se+Si) × ST-III interactions, respectively.

Hydroxytyrosol concentration was drastically higher at ST-I than at ST-II and ST-III. For all the other phenolic compounds reported in Table 2, significant T × ST interactions were determined. Selenium treatment at ST-I, as well as Se+Si treatment at ST-II, resulted in higher oleanolic acid concentrations in olive leaves compared to the control (Table 2). Tyrosol levels did not show unique dynamics within different treatments in olive leaves. The effect of treatment was observable only in the case of higher concentration in Se+Si than in Si at ST-III, while a tendency to higher concentrations toward the latter ST was noted for Se and Se+Si treatments. The lowest tyrosol concentration was recorded at Se × ST-II (Table 2). Regardless of the treatment applied, the highest verbascoside levels were recorded at ST-III. Furthermore, at ST-III, clear statistical differences between all the treatments were documented, with the highest level of 150 mg/100 g DW for Se treatment, followed by the control with 118 mg/100 g DW, Se+Si with 96.3 mg/100 g DW, and Si with 76.7 mg/100 g DW (Table 2).

For all the treatments, higher oleuropein concentrations were recorded at ST-III than at ST-I and ST-II. At ST-III, Si and Se+Si treatments produced higher oleuropein concentrations compared to control, while for Si, this difference was significant at ST-II, as well (Table 2).

Compared to the control treatment, the concentration of the oleuropein-aglycone (monoaldehydic form) was significantly increased only by Se+Si at ST-I (Table 2). On the other hand, treatments that included Si, namely, Se+Si and Si, decreased its concentration at ST-III. In general, the concentration of oleuropein-aglycone increased in the control and Se treatments, and showed a tendency to decrease in the Se+Si and Si treatments at the latest sampling time (ST-III). By contrast, the concentration of a dialdehydic oleuropein aglycone, oleacein was the highest in treatments with Si, namely, Se+Si and Si, at ST-III. Silicon treatment produced a higher oleacein concentration compared to the other treatments at ST-II, as well.

Among the identified flavonoids, the concentration of luteolin-7-O-glucoside increased after Si treatment compared to the control (Table 3). The concentration of luteolin-7-O-glucoside was higher at ST-III compared to the other two STs (Table 3). For other flavonoids, significant T × ST interactions were determined. In most cases, a similar pattern was observed when comparing Se with the control treatment at ST-III, with higher concentrations of rutin, luteolin 4-O-glucoside, luteolin, catechin, and rutin in the former. Besides that, in several cases, the concentrations of flavonoids were increased by different treatments compared to the control, such as apigenin-7-O-glucoside by Se+Si at ST-I and ST-II, luteolin 4-O-glucoside by most treatments at various STs, catechin by Se and Si at ST-I and all treatments at ST-III, as well as rutin by Si at ST-I and all treatments at ST-II. In several cases, for particular flavonoids, an increase in concentration was observed at ST-III.

### 2.2. Concentrations of Elements in Olive Leaves

The concentrations of several elements were significantly affected by T and ST. All the treatments increased the concentration of sodium (Na) compared to the control, with the highest Na concentrations measured in the Si treatment. The concentration of zinc (Zn) was higher in Se+Si than in Se treatments. Expectedly, increased concentrations of Si were found in leaves treated with solution containing Si (both treatments) compared to control and Se treatment (Table 4).

The concentrations of several elements increased toward later sampling times. The increase was linear in the case of calcium (Ca), copper (Cu), Zn, and Si, while for magnesium (Mg), Na, and iron (Fe) it occurred at ST-III. Significant T × S interactions were observed for Se and iodine (I). Selenium treatment increased the concentration of Se element compared to control only at ST-III, while Se+Si treatment caused its increase at each ST. For most treatments, the concentration of I increased at ST-II and decreased at ST-III (Table 4).

### 2.3. Concentrations of Amino Acids, Vitamins, and Other Metabolites in Olive Leaves

The concentration of the ten amino acids determined in olive leaves was affected by T × ST interaction (Table 5). Among them, in ST-III, Si treatment decreased the concentration of arginine (Arg), asparagine (Asn), aspartic acid (Asp), citrulline (Cit), glutamine (Gln), lysine (Lys), Phe, and Trp in comparison to the control treatment. At the same ST, Se treatment, when compared to the control, increased Asn, Gln, histidine (His), Trp, and Tyr concentrations. The increase in amino acid concentrations with time (later STs) exceeded 10-fold, with concentration at ST-III being drastically larger than at the other two STs (Table 5). This effect was also apparent in the T × ST interaction with ST-III, in all instances having the highest concentration of all amino acids measured (Table 5).

Only vitamin E and IAA concentrations were affected by T and ST interaction, while vitamin B2, shikimic acid, and quinic acid concentrations were affected only by STs (Table 6). The concentration of vitamin D2 was not affected by any of the factors. Vitamin E concentration decreased upon every treatment compared to the control at ST-I. The highest concentrations of vitamin B2 and IAA and the lowest concentrations of vitamin E and shikimic acid were measured at ST-III. While vitamin E concentration changes through times within the same treatment were less pronounced (below 2-fold), the increase in IAA concentration in ST-III at each treatment was drastic, with pure Si application resulting in lower IAA concentration compared to Se and control treatments.

### 2.4. Pearson’s Correlation Analysis and Partial Least Squares-Iscriminant Analysis (PLS-DA)

**Figure 1:**
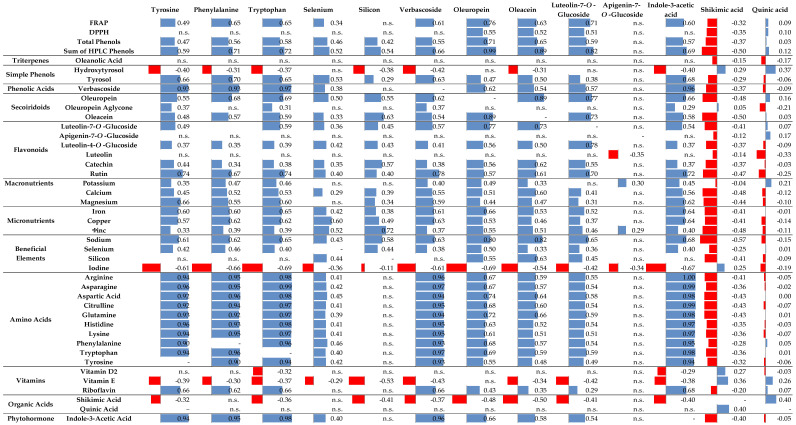
Pearson’s correlation coefficients for tyrosine, phenylalanine, tryptophan, selenium, silicon, verbascoside, oleuropein, oleacein, luteolin-7-O-glucoside, apigenin-7-O-glucoside, indole-3-acetic-acid, shikimic acid, and quinic acid versus all analyzed variables in olive (*Olea europaea* L. Leccino cv.) leaves. Red bars on the left represent a negative, and blue bars on the right represent a positive significant correlation (*p* < 0.05). n.s., not significant.

When significant, correlations between selected dependent variables were mostly strongly positive. However, hydroxytyrosol, I, vitamin E, and shikimic acid showed a predominantly lower degree of negative correlations (Figure 1). By contrast, quinic acid, apigenin-7-O-glucoside, luteolin, and oleanolic acid showed primarily insignificant correlations or very low significant correlations with any of the compounds listed. Metabolically interested positive correlations were, for example, Phe and Trp with verbascoside, total phenols, FRAP, rutin, luteolin-7-O-glucoside, tyrosol and oleuropein aglycone, Trp with IAA, oleuropein and the sum of HPLC phenols, Mg, Na, Fe, and Cu, vitamin B2, and IAA with Trp, Phe, and Tyr. Furthermore, oleuropein correlated positively with oleacein, FRAP, DPPH, and Na. Tyrosine correlated positively with rutin and tyrosol, and negatively with hydroxytyrosol. Zinc, Na, and catechin correlated positively with Si, the same as Zn and Fe with Se. The selected amino acids exhibited significant and strong positive correlations with each AAA and verbascoside.

**Figure 2:**
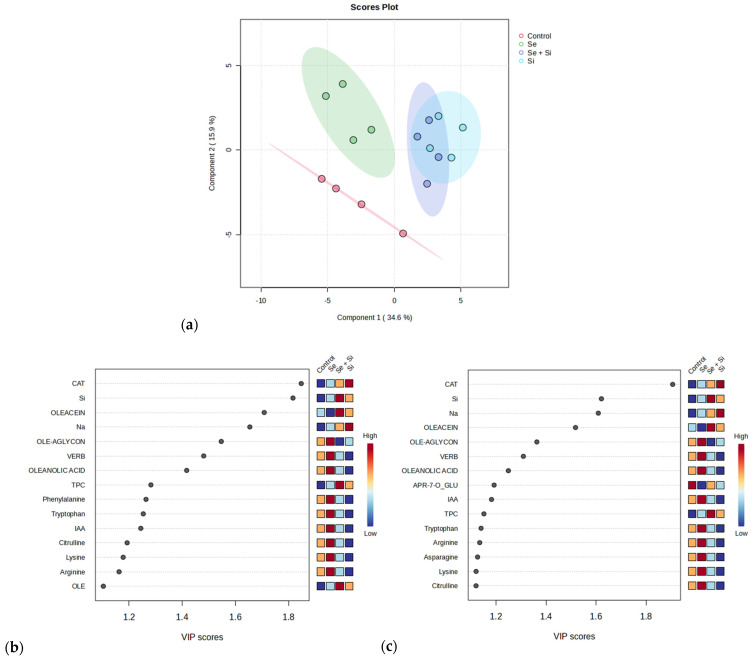
(**a**) Separation of olive leaves at harvest sampling time (ST-III) based on different treatments of foliar fertilization with silicon and selenium in two-dimensional space by partial least squares-iscriminant analysis (PLS-DA); (**b**) variable importance in projection (VIP) scores of variables (phenols, elements, amino acids, and other metabolites) most useful for the separation by component 1; (**c**) variable importance in projection (VIP) scores of variables (phenols, minerals, amino acids, and other metabolites) most useful for the separation by component 2.

In our search to characterize treatment effects in detail at ST-III, PLS-DA was employed, revealing a clear separation of control from foliar-treated plants, with Se treatment grouping away from the Si and Se+Si treatment (Figure 2a). Further analysis indicated that the highest VIP scores for both components explaining 50.5% variance were catechin, Si, Na, and oleacin abundance, which was most characteristic for Si or Se+Si treatments (Figure 2b,c).

**Table 2:** Effect of different foliar treatments, sampling times, and their interactions on triterpenes, simple phenols, phenolic acids, and secoiridoids in olive (*Olea europaea* L. Leccino cv.) leaves treated thrice with a solution of selenium (Se), silicon (Si), or their combination (Se+Si) treatments.

	Triterpenes	Simple Phenols		Phenolic Acids	Secoiridoids		
	Oleanolic Acid	Hydroxytyrosol	Tyrosol	Verbascoside	Oleuropein	Oleuropein Aglycone	Oleacein
	(mg/100 g DW)	(mg/100 g DW)		(mg/100 g DW)	(mg/100 g DW)		
**Treatments (T)**							
Control	517.96 ± 42.23 ^b^	7.69 ± 2.19	18.90 ± 1.17	63.56 ± 11.93 ^ab^	3636.6 ± 341.28 ^c^	11.01 ± 1.31 ^b^	35.85 ± 6.98 ^c^
Se	695.95 ± 47.97 ^a^	10.35 ± 5.30	17.08 ± 1.59	74.98 ± 16.25 ^a^	3936.37 ± 333.09 ^bc^	15.75 ± 1.85 ^a^	34.03 ± 6.92 ^c^
Se+Si	665.38 ± 39.13 ^a^	6.95 ± 1.87	19.93 ± 1.53	53.78 ± 9.22 ^b^	4828.08 ± 461.65 ^a^	10.47 ± 1.84 ^b^	60.72 ± 12.84 ^b^
Si	495.76 ± 54.70 ^b^	5.34 ± 2.05	17.17 ± 0.90	52.96 ± 5.29 ^b^	4661.85 ± 285.31 ^ab^	7.56 ± 1.03 ^b^	76.30 ± 9.78 ^a^
*p*-value	**<0.001**	0.297	0.143	**0.002**	**0.003**	**<0.001**	**<0.001**
**Sampling times (ST)**							
August (ST-I)	518.77 ± 62.49 ^b^	18.71 ± 3.06 ^a^	15.59 ± 0.61 ^b^	31.66 ± 1.23 ^c^	3922.92 ± 134.24 ^b^	10.02 ± 1.10	33.83 ± 2.21 ^b^
September (ST-II)	642.18 ± 35.42 ^a^	2.43 ± 0.71 ^b^	17.25 ± 0.98 ^b^	42.13 ± 1.28 ^b^	3152.91 ± 186.90 ^c^	11.79 ± 0.70	32.23 ± 8.43 ^b^
October (ST-III)	620.34 ± 23.29 ^a^	1.61 ± 0.85 ^b^	21.98 ± 1.17 ^a^	110.18 ± 7.49 ^a^	5721.35 ± 226.58 ^a^	11.79 ± 2.27	89.11 ± 6.57 ^a^
*p*-value	**0.002**	**<0.001**	**<0.001**	**<0.001**	**<0.001**	0.188	**<0.001**
**Treatments × Sampling times**							
Control × ST-I	367.76 ± 46.32 ^de^	16.78 ± 2.48	16.66 ± 1.08 ^bcd^	28.81 ± 0.68 ^e^	3546.85 ± 182.11 ^ef^	7.16 ± 0.77 ^def^	29.89 ± 4.05 ^efg^
Control × ST-II	513.36 ± 28.81 ^cde^	4.25 ± 1.61	18.09 ± 1.89 ^bcd^	43.78 ± 1.84 ^e^	2399.50 ± 81.91 ^g^	9.45 ± 1.15 ^bcdef^	12.08 ± 3.21 ^h^
Control × ST-III	672.76 ± 33.51 ^abc^	2.05 ± 1.43	21.94 ± 2.30 ^abc^	118.10 ± 6.14 ^b^	4963.46 ± 376.31 ^bcd^	16.43 ± 1.20 ^ab^	65.58 ± 3.75 ^c^
Se × ST-I	826.25 ± 114.36 ^a^	30.41 ± 10.38	15.19 ± 0.89 ^cd^	34.04 ± 3.04 ^e^	3622.06 ± 227.95 ^ef^	10.66 ± 1.49 ^bcde^	25.79 ± 2.10 ^efgh^
Se × ST-II	564.93 ± 12.22 ^cd^	0.49 ± 0.09	12.33 ± 1.39 ^d^	41.30 ± 1.88 ^e^	2855.31 ± 141.25 ^fg^	13.38 ± 1.35 ^bcd^	11.48 ± 1.68 ^gh^
Se × ST-III	696.68 ± 26.43 ^abc^	0.15 ± 0.05	23.74 ± 1.26 ^ab^	149.61 ± 9.88 ^a^	5331.74 ± 277.51 ^bc^	23.23 ± 2.10 ^a^	64.81 ± 3.48 ^cd^
(Se+Si) × ST-I	599.76 ± 39.59 ^abcd^	13.95 ± 1.41	15.45 ± 1.71 ^cd^	27.70 ± 1.69 ^e^	4610.61 ± 180.29 ^cde^	14.76 ± 2.85 ^bc^	45.29 ± 2.30 ^de^
(Se+Si) × ST-II	821.73 ± 45.84 ^ab^	3.03 ± 1.56	18.48 ± 1.03 ^abcd^	37.31 ± 2.77 ^e^	3101.20 ± 172.93 ^fg^	13.55 ± 0.72 ^bcd^	18.39 ± 0.76 ^fgh^
(Se+Si) × ST-III	574.64 ± 29.02 ^bcd^	3.89 ± 3.05	25.88 ± 1.59 ^a^	96.33 ± 2.05 ^c^	6772.41 ± 109.40 ^a^	3.10 ± 1.04 ^f^	118.48 ± 4.39 ^a^
Si × ST-I	281.30 ± 34.66 ^e^	13.7 ± 2.91	15.05 ± 1.38 ^cd^	36.08 ± 1.11 ^e^	3912.16 ± 88.38 ^def^	7.48 ± 0.97 ^cdef^	34.35 ± 1.19 ^ef^
Si × ST-II	668.70 ± 59.21 ^abc^	1.95 ± 1.57	20.1 ± 0.96 ^abc^	46.11 ± 2.07 ^e^	4255.61 ± 130.84 ^cde^	10.79 ± 1.47 ^bcde^	86.98 ± 8.05 ^b^
Si × ST-III	537.29 ± 48.29 ^cde^	0.36 ± 0.30	16.36 ± 1.26 ^cd^	76.69 ± 2.04 ^d^	5817.78 ± 427.50 ^ab^	4.41 ± 1.38 ^ef^	107.59 ± 5.88 ^a^
*p*-value	**<0.001**	0.059	**0.001**	**<0.001**	**<0.001**	**<0.001**	**<0.001**

Results are expressed as means ± standard errors (*n* = 4). Different letters for each variable indicate significant differences between mean values for treatment, sampling time, and their interaction (*p* ≤ 0.05) using RM ANOVA and Tukey’s post hoc test (*p* ≤ 0.05), the absence of letters represents an insignificant result.

**Table 3:** Effect of different foliar treatments, sampling times, and their interactions on flavonoids in olive (*Olea europaea* L., Leccino cv.) leaves treated thrice with a solution of selenium (Se), silicon (Si), or their combination (Se+Si).

	Flavonoids					
	Apigenin-7-O-Glucoside	Luteolin-7-O-Glucoside	Luteolin-4-O-Glucoside	Luteolin	Catechin	Rutin
			(mg/100 g DW)			
**Treatments (T)**						
Control	48.95 ± 2.80 ^b^	446.14 ± 30.32 ^b^	23.38 ± 0.93 ^b^	2.86 ± 0.5	21.05 ± 2.65 ^b^	53.64 ± 4.16 ^c^
Se	51.16 ± 1.68 ^b^	543.92 ± 27.57 ^ab^	33.67 ± 1.24 ^a^	3.97 ± 0.52	37.67 ± 2.27 ^a^	70.58 ± 5.67 ^a^
Se+Si	59.81 ± 2.99 ^a^	524.23 ± 45.60 ^ab^	30.15 ± 1.69 ^a^	3.46 ± 0.42	37.81 ± 4.39 ^a^	61.72 ± 5.17 ^b^
Si	49.41 ± 1.31 ^b^	592.04 ± 32.09 ^a^	33.63 ± 0.81 ^a^	3.75 ± 0.42	46.95 ± 3.03 ^a^	69.12 ± 3.65 ^ab^
*p*-value	**0.004**	**0.026**	**<0.001**	0.262	**<0.001**	**<0.001**
**Sampling times (ST)**						
August (ST-I)	55.29 ± 2.73 ^a^	471.54 ± 21.67 ^b^	28.34 ± 1.41 ^b^	2.01 ± 0.22 ^c^	28.36 ± 2.92 ^c^	45.72 ± 1.69 ^c^
September (ST-II)	47.91 ± 1.99 ^b^	473.51 ± 31.01 ^b^	28.74 ± 1.44 ^b^	4.99 ± 0.22 ^a^	33.40 ± 1.93 ^b^	64.56 ± 2.80 ^b^
October (ST-III)	53.80 ± 1.47 ^a^	634.70 ± 24.22 ^a^	33.54 ± 1.27 ^a^	3.53 ± 0.36 ^b^	45.85 ± 4.09 ^a^	81.02 ± 2.46 ^a^
*p*-value	**0.004**	**<0.001**	**<0.001**	**<0.001**	**<0.001**	**<0.001**
**Treatments × Sampling times**						
Control × ST-I	45.40 ± 1.81 ^cd^	406.94 ± 23.59	22.35 ± 0.80 ^de^	1.48 ± 0.27 ^e^	12.88 ± 2.79 ^f^	40.38 ± 2.08 ^f^
Control × ST-II	40.71 ± 2.36 ^d^	357.93 ± 18.34	20.50 ± 0.67 ^e^	4.94 ± 0.52 ^abc^	27.40 ± 4.65 ^de^	48.36 ± 0.64 ^ef^
Control × ST-III	60.75 ± 2.01 ^ab^	573.56 ± 26.42	27.29 ± 0.49 ^cde^	2.18 ± 0.47 ^de^	22.89 ± 3.31 ^ef^	72.19 ± 1.83 ^bcd^
Se × ST-I	54.61 ± 3.16 ^bcd^	489.88 ± 20.00	31.79 ± 1.18 ^abc^	1.76 ± 0.50 ^e^	31.66 ± 3.54 ^cde^	50.14 ± 0.98 ^ef^
Se × ST-II	48.74 ± 3.28 ^bcd^	500.29 ± 24.5	32.24 ± 1.40 ^abc^	5.09 ± 0.41 ^a^	36.58 ± 1.18 ^cde^	68.09 ± 2.76 ^cd^
Se × ST-III	50.13 ± 1.98 ^bcd^	641.59 ± 50.71	36.98 ± 2.84 ^a^	5.05 ± 0.43 ^ab^	44.76 ± 3.69 ^abc^	93.51 ± 5.36 ^a^
(Se+Si) × ST-I	68.88 ± 5.96 ^a^	450.25 ± 62.17	24.50 ± 0.97 ^de^	2.83 ± 0.57 ^bcde^	27.88 ± 2.87 ^def^	39.66 ± 2.36 ^f^
(Se+Si) × ST-II	57.69 ± 2.93 ^abc^	432.16 ± 53.55	28.99 ± 1.65 ^bcd^	4.84 ± 0.55 ^a^	29.18 ± 1.97 ^cdef^	65.39 ± 1.97 ^d^
(Se+Si) × ST-III	52.88 ± 3.06 ^bcd^	690.29 ± 47.98	36.98 ± 1.13 ^a^	2.73 ± 0.62 ^cde^	56.38 ± 5.18 ^ab^	80.11 ± 2.18 ^b^
Si × ST-I	52.26 ± 1.62 ^bcd^	539.10 ± 37.35	34.71 ± 1.53 ^ab^	1.98 ± 0.11 ^e^	41.01 ± 2.15 ^bcd^	52.69 ± 1.37 ^e^
Si × ST-II	44.50 ± 1.02 ^cd^	603.68 ± 67.24	33.25 ± 1.63 ^abc^	5.10 ± 0.43 ^a^	40.46 ± 3.16 ^bcd^	76.41 ± 2.40 ^bcd^
Si × ST-III	51.46 ± 1.72 ^bcd^	633.35 ± 62.18	32.93 ± 1.31 ^abc^	4.18 ± 0.23 ^abcd^	59.36 ± 3.05 ^a^	78.26 ± 1.87 ^bc^
*p*-value	**<0.001**	0.26	**0.002**	**0.002**	**<0.001**	**<0.001**

Results are expressed as means ± standard errors (*n* = 4). Different letters for each variable indicate significant differences between mean values for treatment, sampling time, and their interaction (*p* ≤ 0.05) using RM ANOVA and Tukey’s post hoc test (*p* ≤ 0.05), the absence of letters represents an insignificant result.

**Table 4:** Effect of different foliar treatments, sampling times, and their interactions on minerals in olive (*Olea europaea* L., Leccino cv.) leaves treated thrice with a solution of selenium (Se), silicon (Si), or their combination (Se+Si).

	Macronutrients (g/kg DW)			Micronutrients (mg/kg DW)			Beneficial Elements (mg/kg DW)			
	Potassium	Calcium	Magnesium	Iron	Copper	Zinc	Sodium	Selenium	Silicon	Iodine
**Treatments (T)**										
Control	8.51 ± 0.27	32.63 ± 3.14	1.28 ± 0.04	36.08 ± 1.49	11.08 ± 0.56	19.50 ± 0.77 ^ab^	0.008 ± 0.001 ^c^	0.25 ± 0.03 ^c^	157.83 ± 11.35 ^b^	0.22 ± 0.03
Se	7.84 ± 0.22	29.33 ± 1.84	1.38 ± 0.04	37.24 ± 2.25	10.82 ± 0.67	18.46 ± 0.83 ^b^	0.012 ± 0.002 ^b^	2.84 ± 0.57 ^b^	168.00 ± 15.77 ^b^	0.21 ± 0.04
Se+Si	8.52 ± 0.26	32.85 ± 2.46	1.38 ± 0.04	38.6 ± 2.43	12.66 ± 0.66	24.02 ± 1.34 ^a^	0.014 ± 0.003 ^ab^	5.59 ± 0.74 ^a^	383.84 ± 20.38 ^a^	0.18 ± 0.04
Si	8.18 ± 0.36	33.33 ± 2.32	1.38 ± 0.03	37.08 ± 2.34	10.61 ± 0.57	21.56 ± 0.89 ^ab^	0.017 ± 0.002 ^a^	0.37 ± 0.1 ^c^	370.72 ± 16.67 ^a^	0.25 ± 0.04
*p*-value	0.21	0.262	0.411	0.665	0.053	**0.032**	**<0.001**	**<0.001**	**<0.001**	0.152
**Sampling times (ST)**										
August (ST-I)	8.33 ± 0.25 ^b^	24.37 ± 0.33 ^c^	1.29 ± 0.02 ^b^	34.21 ± 1.14 ^b^	9.32 ± 0.37 ^c^	18.40 ± 0.98 ^c^	0.008 ± 0.001 ^b^	1.27 ± 0.38 ^b^	214.87 ± 28.80 ^c^	0.21 ± 0.02 ^b^
September (ST-II)	7.45 ± 0.13 ^c^	31.45 ± 1.86 ^b^	1.30 ± 0.03 ^b^	33.19 ± 1.65 ^b^	11.12 ± 0.43 ^b^	20.55 ± 0.87 ^b^	0.008 ± 0.001 ^b^	1.78 ± 0.48 ^b^	275.14 ± 28.76 ^b^	0.35 ± 0.02 ^a^
October (ST-III)	9.01 ± 0.18 ^a^	40.28 ± 1.41 ^a^	1.48 ± 0.03 ^a^	44.36 ± 1.15 ^a^	13.46 ± 0.27 ^a^	23.70 ± 0.60 ^a^	0.022 ± 0.002 ^a^	3.73 ± 0.91 ^a^	320.29 ± 29.40 ^a^	0.09 ± 0.01 ^c^
*p*-value	**<0.001**	**<0.001**	**<0.001**	**<0.001**	**<0.001**	**<0.001**	**<0.001**	**<0.001**	**<0.001**	**<0.001**
**Treatments × Sampling times**										
Control × ST-I	8.22 ± 0.50	24.21 ± 0.75	1.22 ± 0.05	30.91 ± 0.64	9.15 ± 0.88	17.03 ± 0.71	0.005 ± 0.000	0.14 ± 0.02 ^e^	113.04 ± 5.62	0.26 ± 0.01 ^ab^
Control × ST-II	7.85 ± 0.20	32.98 ± 7.68	1.19 ± 0.05	36.22 ± 1.95	11.03 ± 0.23	18.85 ± 0.51	0.004 ± 0.000	0.24 ± 0.02 ^e^	158.99 ± 6.38	0.31 ± 0.03 ^ab^
Control × ST-III	9.47 ± 0.23	40.68 ± 1.89	1.44 ± 0.07	41.13 ± 1.68	13.07 ± 0.27	22.62 ± 0.63	0.015 ± 0.001	0.36 ± 0.03 ^e^	201.47 ± 6.44	0.10 ± 0.01 ^cd^
Se × ST-I	7.62 ± 0.20	24.03 ± 0.17	1.33 ± 0.02	32.06 ± 1.01	8.56 ± 0.56	15.88 ± 0.81	0.008 ± 0.000	1.31 ± 0.05 ^e^	106.79 ± 3.56	0.21 ± 0.01 ^bc^
Se × ST-II	7.32 ± 0.28	29.07 ± 2.03	1.29 ± 0.04	34.19 ± 4.43	10.35 ± 0.48	17.74 ± 0.85	0.008 ± 0.000	1.86 ± 0.17 ^de^	175.59 ± 15.49	0.36 ± 0.04 ^a^
Se × ST-III	8.58 ± 0.38	34.9 ± 3.64	1.52 ± 0.07	45.47 ± 0.47	13.56 ± 0.38	21.75 ± 0.51	0.021 ± 0.002	5.35 ± 0.57 ^b^	221.62 ± 16.06	0.07 ± 0.01 ^d^
(Se+Si) × ST-I	9.03 ± 0.32	24.08 ± 0.35	1.31 ± 0.05	38.75 ± 3.05	10.40 ± 0.46	21.14 ± 3.14	0.009 ± 0.001	3.49 ± 0.63 ^cd^	316.29 ± 34.52	0.10 ± 0.01 ^cd^
(Se+Si) × ST-II	7.64 ± 0.21	30.98 ± 0.72	1.39 ± 0.07	31.22 ± 4.02	13.12 ± 1.12	25.21 ± 1.83	0.008 ± 0.001	4.70 ± 0.50 ^bc^	388.72 ± 16.39	0.36 ± 0.03 ^a^
(Se+Si) × ST-III	8.90 ± 0.47	43.49 ± 1.11	1.44 ± 0.07	45.84 ± 1.93	14.47 ± 0.67	25.69 ± 1.52	0.025 ± 0.003	8.58 ± 0.78 ^a^	446.52 ± 16.48	0.09 ± 0.02 ^cd^
Si × ST-I	8.47 ± 0.71	25.15 ± 1.09	1.32 ± 0.04	35.13 ± 1.69	9.15 ± 0.94	19.54 ± 1.58	0.012 ± 0.001	0.16 ± 0.02 ^e^	323.35 ± 25.42	0.27 ± 0.04 ^ab^
Si × ST-II	6.98 ± 0.17	32.78 ± 1.56	1.33 ± 0.03	31.13 ± 2.92	9.97 ± 0.39	20.39 ± 0.28	0.013 ± 0.002	0.32 ± 0.04 ^e^	377.27 ± 20.53	0.37 ± 0.05 ^a^
Si × ST-III	9.08 ± 0.32	42.06 ± 2.74	1.50 ± 0.06	45.00 ± 3.85	12.72 ± 0.44	24.74 ± 0.96	0.027 ± 0.003	0.63 ± 0.26 ^e^	411.54 ± 25.93	0.12 ± 0.03 ^cd^
*p*-value	0.301	0.799	0.28	0.283	0.444	0.486	0.232	**<0.001**	0.905	**0.006**

Results are expressed as means ± standard errors (*n* = 4). Different letters for each variable indicate significant differences between mean values for treatment, sampling time, and their interaction (*p* ≤ 0.05) using RM ANOVA and Tukey’s post hoc test (*p* ≤ 0.05), the absence of letters represents an insignificant result.

**Table 5:** Effect of different foliar treatments, sampling times and their interactions on amino acids in olive (*Olea europaea* L., Leccino cv.) leaves treated thrice with a solution of selenium (Se), silicon (Si) or their combination (Se+Si).

	mg/100 g DW									
	Arginine	Asparagine	Aspartic Acid	Citrulline	Glutamine	Histidine	Lysine	Phenylalanine	Tryptophan	Tyrosine
**Treatments (T)**										
Control	9.609 ± 3.503 ^ab^	2.991 ± 1.255 ^ab^	2.718 ± 0.912 ^ab^	1.159 ± 0.43 ^ab^	26.862 ± 9.647	0.601 ± 0.257 ^ab^	0.575 ± 0.195 ^ab^	0.966 ± 0.267 ^a^	5.556 ± 1.839 ^ab^	2.32 ± 0.744 ^ab^
Se	11.625 ± 4.304 ^a^	4.203 ± 1.683 ^a^	3.222 ± 1.126 ^a^	1.373 ± 0.508 ^a^	35.557 ± 13.363	0.982 ± 0.393 ^a^	0.758 ± 0.268 ^a^	0.997 ± 0.277 ^a^	6.97 ± 2.392 ^a^	3.9 ± 1.22 ^a^
Se+Si	8.056 ± 2.862 ^b^	2.662 ± 1.025 ^ab^	2.66 ± 0.928 ^ab^	0.994 ± 0.35 ^b^	25.517 ± 9.433	0.525 ± 0.196 ^ab^	0.468 ± 0.155 ^ab^	0.882 ± 0.264 ^ab^	4.633 ± 1.529 ^b^	2.237 ± 0.746 ^ab^
Si	6.784 ± 2.229 ^b^	2.034 ± 0.746 ^b^	2.04 ± 0.63 ^c^	0.822 ± 0.272 ^b^	26.946 ± 9.354	0.406 ± 0.14 ^c^	0.342 ± 0.083 ^b^	0.538 ± 0.109 ^b^	3.86 ± 1.064 ^b^	1.886 ± 0.444 ^b^
*p*-value	**0.008**	**0.012**	**0.025**	**0.008**	**0.074**	**0.019**	**0.017**	**0.029**	**0.007**	**0.025**
**Sampling times (ST)**										
August (ST-I)	1.077 ± 0.139 ^b^	0.297 ± 0.042 ^b^	0.71 ± 0.06 ^b^	0.148 ± 0.016 ^b^	3.533 ± 0.299 ^b^	0.084 ± 0.007 ^b^	0.144 ± 0.012 ^b^	0.366 ± 0.024 ^b^	1.362 ± 0.096 ^b^	0.613 ± 0.092 ^b^
September (ST-II)	2.283 ± 0.342 ^b^	0.297 ± 0.037 ^b^	0.485 ± 0.035 ^b^	0.25 ± 0.037 ^b^	6.106 ± 0.712 ^b^	0.083 ± 0.007 ^b^	0.153 ± 0.017 ^b^	0.316 ± 0.019 ^b^	1.404 ± 0.099 ^b^	1.218 ± 0.168 ^b^
October (ST-III)	23.696 ± 1.762 ^a^	8.323 ± 0.753 ^a^	6.785 ± 0.417 ^a^	2.864 ± 0.205 ^a^	76.522 ± 4.508 ^a^	1.719 ± 0.209 ^a^	1.31 ± 0.147 ^a^	1.856 ± 0.173 ^a^	12.997 ± 1.082 ^a^	5.927 ± 0.702 ^a^
*p*-value	**<0.001**	**<0.001**	**<0.001**	**<0.001**	**<0.001**	**<0.001**	**<0.001**	**<0.001**	**<0.001**	**<0.001**
**Treatments × Sampling times**										
Control × ST-I	1.171 ± 0.148 ^d^	0.283 ± 0.032 ^d^	0.741 ± 0.05 ^c^	0.164 ± 0.034 ^d^	3.789 ± 0.261 ^c^	0.08 ± 0.009 ^c^	0.158 ± 0.009 ^de^	0.443 ± 0.015 ^b^	1.535 ± 0.239 ^d^	0.656 ± 0.235 ^d^
Control × ST-II	3.023 ± 1.319 ^d^	0.331 ± 0.117 ^d^	0.566 ± 0.098 ^c^	0.292 ± 0.143 ^d^	7.572 ± 2.532 ^c^	0.087 ± 0.025 ^c^	0.184 ± 0.058 ^de^	0.354 ± 0.037 ^b^	1.334 ± 0.235 ^d^	1.028 ± 0.436 ^cd^
Control × ST-III	24.631 ± 4.452 ^ab^	8.358 ± 1.705 ^b^	6.847 ± 0.784 ^a^	3.022 ± 0.528 ^ab^	69.226 ± 10.84 ^b^	1.637 ± 0.432 ^b^	1.384 ± 0.296 ^ab^	2.1 ± 0.373 ^a^	13.797 ± 1.771 ^b^	5.275 ± 1.205 ^b^
Se × ST-I	1.171 ± 0.358 ^d^	0.337 ± 0.096 ^d^	0.789 ± 0.153 ^c^	0.156 ± 0.041 ^d^	4.016 ± 0.863 ^c^	0.101 ± 0.004 ^c^	0.167 ± 0.028 ^de^	0.434 ± 0.048 ^b^	1.413 ± 0.186 ^d^	0.531 ± 0.234 ^d^
Se × ST-II	2.001 ± 0.326 ^d^	0.225 ± 0.04 ^d^	0.401 ± 0.032 ^c^	0.229 ± 0.049 ^d^	5.493 ± 0.751 ^c^	0.071 ± 0.009 ^c^	0.136 ± 0.019 ^de^	0.3 ± 0.035 ^b^	1.527 ± 0.165 ^d^	1.879 ± 0.241 ^cd^
Se × ST-III	31.704 ± 1.362 ^a^	12.047 ± 0.622 ^a^	8.475 ± 0.328 ^a^	3.736 ± 0.201 ^a^	97.163 ± 8.057 ^a^	2.774 ± 0.312 ^a^	1.97 ± 0.235 ^a^	2.257 ± 0.214 ^a^	17.97 ± 1.55 ^a^	9.29 ± 1.196 ^a^
(Se+Si) × ST-I	0.989 ± 0.306 ^d^	0.288 ± 0.095 ^d^	0.702 ± 0.117 ^c^	0.133 ± 0.029 ^d^	3.218 ± 0.5 ^c^	0.1 ± 0.019 ^c^	0.12 ± 0.022 ^e^	0.29 ± 0.02 ^b^	1.359 ± 0.134 ^d^	0.499 ± 0.139 ^d^
(Se+Si) × ST-II	1.911 ± 0.404 ^d^	0.328 ± 0.089 ^d^	0.428 ± 0.076 ^c^	0.239 ± 0.05 ^d^	4.334 ± 0.741 ^c^	0.074 ± 0.012 ^c^	0.124 ± 0.027 ^e^	0.286 ± 0.05 ^b^	1.025 ± 0.13 ^d^	0.878 ± 0.281 ^cd^
(Se+Si) × ST-III	21.268 ± 1.554 ^bc^	7.371 ± 0.677 ^bc^	6.851 ± 0.813 ^a^	2.609 ± 0.186 ^bc^	68.999 ± 5.702 ^b^	1.401 ± 0.199 ^b^	1.159 ± 0.151 ^bc^	2.07 ± 0.235 ^a^	11.514 ± 1.405 ^bc^	5.335 ± 1.099 ^b^
Si × ST-I	0.978 ± 0.36 ^d^	0.28 ± 0.123 ^d^	0.607 ± 0.163 ^c^	0.138 ± 0.038 ^d^	3.108 ± 0.732 ^c^	0.057 ± 0.015 ^c^	0.13 ± 0.034 ^de^	0.298 ± 0.035 ^b^	1.141 ± 0.214 ^d^	0.763 ± 0.156 ^cd^
Si × ST-II	2.195 ± 0.258 ^d^	0.304 ± 0.046 ^d^	0.543 ± 0.029 ^c^	0.239 ± 0.03 ^d^	7.028 ± 0.664 ^c^	0.098 ± 0.009 ^c^	0.169 ± 0.017 ^de^	0.321 ± 0.033 ^b^	1.732 ± 0.068 ^d^	1.089 ± 0.184 ^cd^
Si × ST-III	17.179 ± 0.419 ^c^	5.517 ± 0.187 ^c^	4.97 ± 0.208 ^b^	2.09 ± 0.082 ^c^	70.702 ± 1.274 ^b^	1.063 ± 0.02 ^b^	0.728 ± 0.024 ^cd^	0.996 ± 0.152 ^b^	8.706 ± 0.767 ^c^	3.807 ± 0.499 ^bc^
*p*-value	**0.002**	**<0.001**	**0.002**	**0.005**	**0.017**	**<0.001**	**0.001**	**0.003**	**<0.001**	**0.006**

Results are expressed as means ± standard errors (*n* = 4). Different letters for each variable indicate significant differences between mean values for treatment, sampling time, and their interaction (*p* ≤ 0.05) using RM ANOVA and Tukey’s post hoc test (*p* ≤ 0.05), the absence of letters represents an insignificant result.

**Table 6:** Effect of different foliar treatments, sampling times, and their interactions on other metabolites in olive (*Olea europaea* L., Leccino cv.) leaves treated thrice with a solution of selenium (Se), silicon (Si), or their combination (Se+Si).

	mg/100 g DW					
	Vitamin D2	Vitamin E	Vitamin B2	Shikimic Acid	Quinic Acid	Indole-3-Acetic Acid
**Treatments (T)**						
Control	4.813 ± 0.328	1.956 ± 0.172 ^a^	7.907 ± 0.817	5.901 ± 0.302	2808.469 ± 91.924	24.798 ± 8.448 ^ab^
Se	4.527 ± 0.36	1.582 ± 0.072 ^b^	9.632 ± 1.13	5.521 ± 0.279	2610.986 ± 128.658	28.837 ± 10.269 ^a^
Se+Si	4.296 ± 0.413	1.535 ± 0.051 ^bc^	7.609 ± 0.85	5.294 ± 0.377	2818.841 ± 169.285	19.936 ± 6.723 ^bc^
Si	4.964 ± 0.524	1.445 ± 0.03 ^c^	7.109 ± 0.679	5.2 ± 0.233	2633.679 ± 150.637	16.54 ± 5.193 ^c^
*p*-value	0.658	**<0.001**	0.207	0.464	0.696	**0.004**
**Sampling times (ST)**						
August (ST-I)	5.009 ± 0.409	1.976 ± 0.121 ^a^	7.496 ± 0.421 ^b^	6.022 ± 0.204 ^a^	2946.443 ± 115.296 ^a^	3.956 ± 0.444 ^b^
September (ST-II)	4.886 ± 0.232	1.498 ± 0.037 ^b^	5.916 ± 0.394 ^b^	5.674 ± 0.227 ^a^	2489.202 ± 109.123 ^b^	6.316 ± 0.906 ^b^
October (ST-III)	4.055 ± 0.363	1.414 ± 0.023 ^c^	10.781 ± 0.864 ^a^	4.74 ± 0.253 ^b^	2718.336 ± 108.026 ^ab^	57.311 ± 4.374 ^a^
*p*-value	0.166	**<0.001**	**<0.001**	**0.002**	**0.007**	**<0.001**
**Treatments × Sampling times**						
Control × ST-I	5.537 ± 0.742	2.736 ± 0.07 ^a^	7.906 ± 0.677	6.481 ± 0.371	3111.442 ± 193.066	5.001 ± 0.95 ^d^
Control × ST-II	4.824 ± 0.398	1.698 ± 0.068 ^bcd^	5.752 ± 1.087	6.161 ± 0.501	2696.097 ± 48.725	8.366 ± 3.475 ^d^
Control × ST-III	4.079 ± 0.348	1.433 ± 0.019 ^ef^	10.065 ± 1.612	5.059 ± 0.492	2617.87 ± 79.908	61.028 ± 10.673 ^ab^
Se × ST-I	4.752 ± 0.346	1.893 ± 0.061 ^b^	9.239 ± 0.656	5.873 ± 0.431	2908.895 ± 139.52	4.361 ± 0.897 ^d^
Se × ST-II	4.915 ± 0.597	1.451 ± 0.053 ^def^	5.746 ± 0.901	5.697 ± 0.284	2388.597 ± 221.086	5.344 ± 0.513 ^d^
Se × ST-III	3.913 ± 0.871	1.402 ± 0.041 ^ef^	13.912 ± 1.27	4.993 ± 0.669	2535.465 ± 256.449	76.806 ± 2.877 ^a^
(Se+Si) × ST-I	3.784 ± 0.447	1.729 ± 0.058 ^bc^	6.849 ± 0.703	5.857 ± 0.605	3019.136 ± 422.919	3.517 ± 0.781 ^d^
(Se+Si) × ST-II	4.583 ± 0.586	1.393 ± 0.027 ^ef^	6.3 ± 0.718	5.47 ± 0.74	2561.562 ± 297.753	5.543 ± 1.089 ^d^
(Se+Si) × ST-III	4.52 ± 1.094	1.482 ± 0.066 ^def^	9.679 ± 2.179	4.554 ± 0.59	2875.825 ± 105.814	50.747 ± 4.474 ^bc^
Si × ST-I	5.961 ± 1.248	1.545 ± 0.044 ^cde^	5.989 ± 0.444	5.877 ± 0.217	2746.3 ± 74.854	2.942 ± 0.893 ^d^
Si × ST-II	5.221 ± 0.384	1.451 ± 0.02 ^def^	5.868 ± 0.729	5.369 ± 0.168	2310.552 ± 261.187	6.012 ± 0.975 ^d^
Si × ST-III	3.71 ± 0.658	1.34 ± 0.025 ^f^	9.469 ± 1.249	4.356 ± 0.352	2844.183 ± 349.261	40.665 ± 1.532 ^c^
*p*-value	0.611	**<0.001**	0.250	0.996	0.665	**0.002**

Results are expressed as means ± standard errors (*n* = 4). Different letters for each variable indicate significant differences between mean values for treatment, sampling time, and their interaction (*p* ≤ 0.05) using RM ANOVA and Tukey’s post hoc test (*p* ≤ 0.05), the absence of letters represents an insignificant result.

## 3. Discussion

The role of mineral nutrition in olive leaves secondary metabolism has been overlooked until recently, with only a few studies conducted on the subject [31]. In the light of olive leaves being recognized as a valuable raw material, particularly due to their secondary metabolite composition, the topic is of great interest. Particularly as the synthesis of secondary metabolites, such as phenolic compounds, has been shown to be affected by a variety of abiotic factors, including fertilization. Therefore, the changes in phenolic profile, both qualitative and quantitative, can be expected within specific foliar nutrient applications [32].

### 3.1. Amino Acids and Other Metabolites

Phosphoenolpyruvate (PEP) and erythrose-4-phosphate (E4P) are formed in glycolysis and in the pentose phosphate pathway, respectively, initiating the shikimate pathway. In this metabolic route, with shikimic acid as an important intermediate, AAAs are produced [33]. Quinic acid is another intermediate product of the shikimate pathway and can be interconverted with shikimic acid, serving as a storage form for important intermediates. Among AAAs, Phe and Tyr are defined as those that are connected to different routes of olive phenolic compound biosynthesis [5].

The highest amino acid concentrations in olive leaves of all treatments were recorded at the harvest sampling time, ST-III. At ST-III, Si treatment had a negative impact on seven amino acids and IAA levels in comparison with the control (Table 5 and Table 6). On the contrary, Se treatment increased the levels of five amino acids including Trp and Tyr (Table 6). Ježek et al. [34] noticed an increase in the content of essential and non-essential amino acids in potatoes after foliar Se application, with higher Phe and Tyr concentrations than in a control treatment. Strawberry fruits treated with Se exhibited a significant accumulation of Phe [35]. Total free amino acid levels were increased by Si application on squash plants [36]. The concentrations of shikimic and quinic acid, the precursors of AAAs, along with vitamin E, were the highest at ST-I compared to other sampling times. Among them, control treatment had a clear tendency only toward higher levels of vitamin E in ST-I (Table 6), leaving it unclear to draw simple conclusions from available information.

### 3.2. Antioxidant Capacity, Total Phenols, and Sum of HPLC Phenols and Their Relationship with Aromatic Amino Acids

Olive leaf antioxidant potential has been confirmed in various studies and was often cultivar-specific [37,38,39] and temporal-dependent [2,40]. Sadati et al. [41] reported a beneficial impact of foliar-sprayed potassium sulfate on the antioxidant activity in olive leaves, while Graziani et al. [42] noted that the use of different biostimulants on young olive plants resulted in higher DPPH and FRAP values and total phenolic content in leaves. In a recent study, Si fertilization did not influence the antioxidant capacity of peach and nectarine fruits [43] but resulted in higher levels of total and specific phenolic compounds and the antioxidant capacity of common buckwheat [44]. The results of this study confirmed that FRAP values, total phenol concentration, and the sum of HPLC phenols were dependent on the sampling time (Table 1) and were strongly correlated with Phe, Trp, and oleuropein concentrations (Figure 1). The connection of antioxidative capacity and phenols concentration with oleuropein in olive leaves was expected as oleuropein is the main phenolic compound with proven antioxidant capacity in olives. De Bruno et al. [22] tested table olive products and reported increased antioxidant activity after Se foliar field application. Moreover, in an experiment with rabbits fed with Se-enriched olive leaves, better meat oxidative status was observed when compared to meat from rabbits on other diets, confirming the potential of such treated leaves for increasing the antioxidant capacity of foods [45,46]. According to D’Amato et al. [23], EVOO obtained from olive trees fertilized with Se had greater oxidative stability and longer shelf life. Additionally, the foliar application of selenate in an open field trial increased the antioxidant activity of tea extracts, in comparison with selenite fertilization and conventional tea cultivation methods [47]. Xu et al. [47] found that applying selenate fertilizer at a concentration of 60 mg/L Se led to tea leaf sprouts with higher Se concentration (10.6 mg/kg DW) and lower total phenolic content compared to an untreated control. Although in this study such a difference in total phenols between the treatments was not recorded, the sum of HPLC phenols in Si+Se and Si treatments was shown to be higher than that in control treatment at almost each sampling time when considering T × ST interactions (Table 1).

Tarasevičine et al. [48] reported that foliar Trp application, compared with a water-sprayed control, increased the concentration of total phenols in mint. As Trp is a precursor for IAA biosynthesis, such a result implied a potential interplay between IAA, as an auxin, and phenolics in plant stress response. Aromatic amino acids such as Phe, Trp, and Tyr play a significant role in the biosynthesis of secondary metabolites in plants [49], so it was not surprising that the mentioned AAAs showed a strong positive correlation with the sum of HPLC phenolics across the whole dataset (Figure 1). This was probably strongly related to the increase observed at ST-III for both AAAs and the sum of HPLC phenols. However, a direct connection between the levels of Phe, Trp, and Tyr (Table 6) and those of total phenols and the sum of HPLC phenols (Table 1) was not evident, since the effects of foliar treatments with Se, Si+Se, and Si on these two groups of compounds did not coincide. A similar relationship was observed between IAA and the sum of HPLC phenols.

### 3.3. Simple Phenols, Phenolic Acids, Flavonoids, and Their Relationship with Amino Acids

Tyrosol and hydroxytyrosol belong to a group of plant phenolic compounds called phenylethanoids [50]. While it has been previously reported that tyrosol is synthesized from Tyr, the biosynthesis of hydroxytyrosol, a simple orto-diphenol, involves a complex network of enzymes and is still not fully understood [50,51,52]. However, recent findings on young olive fruits confirmed that Tyr may serve as a precursor to hydroxytyrosol in two different biosynthetic pathways [52]. In this study, a moderate negative correlation between Tyr and hydroxytyrosol was observed. It seems that their relationship cannot be interpreted solely on the basis of correlation analysis, since hydroxytyrosol biosynthesis was probably affected by a complex interplay of various enzymes and an array of substrates and intermediates. Simple phenols hydroxytyrosol and tyrosol showed mostly temporal variations from August (ST-I) to October (ST-III) sampling times (Table 2). Pasković et al. [3] noticed such variations in Leccino cultivar olive leaves between harvest, winter dormancy, and pruning periods. Hydroxytyrosol and tyrosol were not affected by the applied treatments (Table 2). However, in a study with hydroponically grown strawberries, the highest, up to a 2000-fold increase in tyrosol fruit concentration was recorded when 100 µM Se-treated plants were compared to the control ones [35]. The study conducted by Gomes et al. [53] discovered that using Na metasilicate in a foliar treatment in vineyards led to a reduction in the concentration of tyrosol in produced wine.

Plants produce a wide range of phenylpropanoids, such as flavonoids and phenolic acids (benzoic and hydroxycinnamic acids and their derivatives), which are derived from Phe through the phenylpropanoid metabolism [54]. Among them, verbascoside is the main hydroxycinnamic derivative in olives [55]. Our data revealed a very strong positive correlation (*r* > 0.90) between verbascoside and Phe, but also Tyr, Trp, and other amino acids, such as Asp, Asp, Gln, Cit, Lys, (His), and Arg. In ST-III, Se treatment increased verbascoside concentration (Table 2), which closely aligned to the increased levels of Trp and Tyr in Se × III compared to Control × III (Table 5). Also, the level of IAA showed a tendency to be higher in Se × III than in Control × III (Table 6). On the other hand, considering only the data obtained at ST-III, Si exhibited a strong negative correlation with verbascoside (*r* = −0.76, *p* = 0.001), indicating that Se-Si interplay could be substantial for olive metabolic response regarding the synthesis of verbascoside, and possibly stress. Se × Si and especially Si treatment significantly reduced the concentration of verbascoside in ST-III (Table 2), which again coincided with a tendency of Trp, Tyr, and IAA to have lower levels in leaves of these treatments. Although the connection of verbascoside with AAAs such as Trp and Tyr, as well as with IAA, seemed obvious, such results have to be considered with caution. Rosato et al. [56] emphasized that a strong positive correlation between two metabolites can be connected to a rapid equilibrium or the dominance of an enzyme involved in the biosynthetic step in question, but at the same time, a strong correlation can be recorded between components that are not metabolic neighbors.

The accumulation of verbascoside in plant tissues, along with flavonoids such as apigenin-7-O-glucoside and luteolin-7-O-glucoside, has been previously connected with their specific role in olive metabolism response against stress conditions [57]. In this study, flavonoid glucosides have shown a tendency toward higher concentrations under Se+Si and Si foliar fertilization treatments (Table 3). Significant positive correlations of two luteolin glucosides, luteolin-7-O-glucoside and luteolin-4-O-glucoside, with AAAs across the whole dataset (Figure 1) suggest a possibility of a certain amplification of the biosynthetic pathways in which they participate in a precursor–product relationship as a response to the treatments with Si and Se, although this connection was not as clear as in the case of verbascoside. Plants use flavonoids as a secondary system to neutralize reactive oxygen species and prevent cell damage caused by radicals [57]. In healthy leaf cells, glycosylated flavonoids are frequently detected and have the potential to perform reducing activity [58]. Catechin has been proven as one of the most active agents in the reduction of fungal growth under in vitro conditions and may play an important role in the olive plant fungal pathogen defense system [59]. In this study, most of the foliar treatments increased luteolin-4-O-glucoside, catechin, and rutin concentrations, depending on the ST (Table 3).

### 3.4. Triterpenes and Their Relationship with Selected Amino Acids

Terpenes or isoprenes, including triterpenes (C30), encompass a diverse and complex group of hydrocarbons with a repeated presence of a basic skeleton of the C5 isoprene unit in their structure. They are natural compounds vital for plants, participating in their primary metabolism as phytohormones. However, they are mostly involved in plant secondary metabolism due to their roles as stress-response molecules [60]. The predominant triterpene found in olive leaves is oleanolic acid [61]. Triterpenoids, such as oleanolic acid in *Ligustrum lucidi* fruits, which also belong to the *Oleaecea* family, seem to be synthesized from the compounds that are generated through both the cytosolic mevalonic acid (MVA) pathway and the plastidial methylerythritol phosphate (MEP) pathway [62]. Universal C5 isoprenoid precursors, isopentenyl diphosphate (IPP), and dimethylallyl diphosphate (DMAPP), which are involved in geraniol biosynthesis, can be synthesized from both pathways [62,63]. Oleuropein biosynthesis in olives, on the other hand, seems to be connected to the MVA pathway [54]. In this study, no significant correlations of oleanolic acid with oleuropein and AAA in the investigated olive leaves were found (Figure 1).

In our previous research related to Mn olive nutrition, a higher concentration of oleanolic acid was detected in OLs under Mn-deficient olive plantlets [64]. In this study, under optimal Mn concentration ranges for olive leaves, different Se treatments resulted in nonuniform oleanolic acid concentration changes during the three sampling times (ST-I to ST-III). It seems that foliar treatment with Se may have the ability to modulate the content of this pentacyclic triterpenoid in olive leaves. Guinda et al. [61] observed a negative correlation between the concentration of oleanolic acid in olive fruit and the number of eggs laid by the olive fly. The authors also suggested that triterpenes, specifically oleanolic acid found in olive leaves, may have a crucial role in defense against pathogenic microorganisms that gain access to the plant through natural openings such as stomata.

### 3.5. Secoiridoids and Their Relationship with Aromatic Amino Acids

Plants use a range of secondary metabolites as a natural defense system against biotic and abiotic stress. It is well known that different plants share common secondary metabolites, but olives are characterized by a unique set of phenols from the group of secoiridoids, called oleosides, that are only found in the Oleaceae family [65]. Among them, oleuropein is the most abundant, representing up to 85% of total olive leaf phenolic compounds [66], and it is, as are other oleosides, a result of a merging of terpene synthesis via the MVA pathway and phenylpropanoid metabolism [54]. In contrast to oleanolic acid, which is also connected to MVA metabolism, oleuropein exhibited strong correlations with all the selected amino acids in this study (Figure 1). Conversely, oleuropein, as an ester of elenolic acid and hydroxytyrosol [54], did not correlate with its precursor, hydroxytyrosol. The proposed biotransformation pathways of oleuropein to the other two secoiridoids identified in this study have been described as a one-step transformation into oleuropein aglycone (3,4-DHPEA-EA) and a five-step transformation (via oleuropein aglycone as an intermediate product) into oleacein (3,4-DHPEA-EDA), respectively [67]. Oleacein has been pointed out as one of the major phenolic compounds identified in virgin olive oil, with confirmed beneficial effects on human health [68]. In a previous study, Se foliar fertilization resulted in increased concentrations of phenolic compounds such as oleacein, ligstroside aglycone, and oleocanthal in virgin olive oil for 32% to 57% compared to a control [69]. In this study, oleuropein and oleacein concentrations have shown a very strong positive mutual correlation in olive leaves (Figure 1, suggesting a direct positive precursor–product relationship. In olive leaves of all the applied treatments, an increase in oleuropein and oleacein concentrations was observed at ST-III (Table 2). The same trend was noted for the control, which suggests that this increase was at least partly a result of normal phenol metabolism during plant development. The Se+Si combined application resulted in a significant increase in oleuropein and oleacein concentration in olive leaves at harvest time ST-III (Table 2), confirming the biostimulant nature of their combined activities. Oleuropein aglycon showed a different behavior: Control and Se treatments increased their concentration at ST-III, but Si and especially Se+Si reduced it notably. It was assumed that Si boosted oleuropein aglycon transformation into oleacein, which was corroborated by higher concentrations of oleacein in Si and Se+Si compared to control and Se treatments in olive leaves at ST-III (Table 2). In general, judging from the PLS-DA results, it can be stated that Si supplementation produced a more distinguishable effect on the phenolic composition of olive leaves, especially catechin and secoiridoids, since these compounds were the most useful for the differentiation of treatments, with a clearer separation of Se+Si and Si from the others (Figure 2).

Significant positive correlations between the levels of oleuropein and oleacein with those of AAAs (Figure 1) were probably largely a result of their joint increase in ST-III olive leaves of all treatments, including the control one (Table 2 and Table 5). On the other hand, a similarity in the trends of the changes in their concentrations as a result of the applied treatments, as noted in the case of verbascoside, was not observed. It is possible that the two branches in the initial stages of the synthesis of secoiridoids, the MVA and phenylpropanoid pathways, responded differently to the applied treatments, meaning the synthesis and metabolism of AAAs was only a factor among others that possibly did not prevail in the determination of final secoiridoid concentrations in olive leaves.

### 3.6. Element Composition

Silicon essentiality as a nutrient for olive plant growth has not been proven yet, but at certain concentrations, it can enhance nutrient uptake and translocation, particularly of potassium (K) [25]. In this experiment, such changes in K concentration were not recorded in any of the Si-based treatments and were only dependent on sampling time (Table 4). Furthermore, it is known that fertilization of plants with Se can influence the absorption and accumulation of other essential minerals related to plant metabolism [70]. Thus, implementing plant nutrition-based biofortification strategies for seven mineral elements that are commonly lacking in the human diet, Fe, Zn, Cu, Ca, Mg, Se, and I, may hold great potential for resolving mineral malnutrition in humans [71].

Of all the previously mentioned minerals essential for the human diet, only Se-containing treatments increased Se concentration in olive leaves, with the highest total Se levels achieved by Se+Si foliar treatment (Table 4), It is possible that silicate anions altered the ion balance in the leaves and/or changed the physico-chemical properties of the nutrient solution remaining on the leaf surface, thereby affecting the uptake of selenate. However, the aforementioned point remains speculative, as no evidence to confirm the interactions between the two anions in this context was found in the available literature. Interestingly, a similar phenomenon was previously observed by Hussain et al. [72] following specific treatments with combined foliar applications of Se and Si compared to Se alone in rice. Our sampled leaves showed a rather significant increase in Se and Si concentrations, likely due in part to the treatments being applied three times: the consistent accumulation of both elements was observed from SP-I to SP-III. As well, the sampled leaves were relatively young, and their thinner cuticles [73] likely facilitated nutrient absorption. Nonetheless, it remains uncertain whether the increase in Si and Se concentrations is exclusively attributable to their penetration through the epidermis and subsequent entry into inner leaf tissues. Further investigation is needed to determine the precise tissue localization of foliar-applied Si and Se in olive leaves, aligning with the tissue-specific mineral allocation study in olive leaves previously reported by Pongrac et al. [74].

When high levels of inorganic Se are applied to plants, they can metabolize and accumulate Se in various organic derivative forms, which reduces its toxicity. When these forms are bioaccumulated in edible plant tissues, such Se-enriched foods can be used as nutraceuticals for humans and animals [23]. Although some previous studies indicated that large amounts of foliar sprayed Se are transformed in plants into organic Se compounds, this transformation depends on plant species and tissues [23,75,76]. However, the intricate nature of Se chemistry in the environment and in different living organisms, including plants, unquestionably presents substantial analytical challenges [77,78]. As a result, many studies that have previously studied combined Se and Si applications on different crops have been focused solely on the total Se content [79,80,81,82,83,84,85,86,87,88,89].

In general, higher concentrations of Se and Si in olive leaves of the corresponding treatments (Table 4) were, as expected, a direct result of their application. There have been multiple studies about the use of Na selenate on olives, and none of them have stressed any negative health risk of such application [22,23,28,45,46,69,90,91,92,93]. These studies found that the total Se levels in olive leaves can range up to 4.8 mg/kg DW [78,80]. Additionally, Mattioli et al. [45,46,90] reported that dried olive leaves enriched with Se (2.17 mg/kg DW), produced by foliar application of Na selenate (100 mg/L Se) in an olive grove, can be safely used (at a 10% contribution) in the feed of rabbits.

No safety concerns are anticipated when inorganic and organic Se compounds are added to feed at levels up to the maximum total Se amount permitted in the EU (0.5 mg/kg). There are additional criteria for organic Se, which should not exceed 0.2 mg/kg in feed [94]. Furthermore, according to EU regulation, Na selenate, along with other Se substances such as Na selenite, Na hydrogen selenite, L-selenomethionine, and Se-enriched yeasts, may be included in food and food supplements [95]. The no-observed-adverse-effect level (NOAEL) for both Na selenate and selenite was defined in rats at 0.4 mg Se/kg body weight [96]. The estimated acute oral toxicity level of Na selenate in rats is 7 mg/kg; while the toxicity for fish, determined by flow-through test LC50, is defined at 2.06 mg/L for 96 h [97]. Nevertheless, further studies are needed to elucidate the transformations of different Se forms in foliar-treated olive leaves if they are used as infusions or related olive leaf products.

Broadley et al. [17] compared Si concentration in the shoots of different plant species, such as rice, wheat, pumpkin, zucchini, chickpea, cucumber, or maize, ranging from 3000 to 39100 mg/kg DW. Hodson et al. [98] reported an average Si concentration of 320 mg/kg in non-woody shoot tissues of *Olea europaea* L. plants. Thus, our data did not reveal any extensive Si accumulation in olive leaves with the highest levels up to 446 mg/kg DW in Si-treated olive leaves (Table 4). These values are in accordance with previously published Si research on Arbequina and Picual cultivars by Nascimento-Silva et al. [99] or Leccino and Istarska Bjelica cultivars by Pasković and Franić et al. [100].

The levels of the other mineral elements detected in olive leaves in this study were found to be in an optimum range, according to Therios [101], with the exception of Fe, for which a relative deficiency can be presumed. At the harvest sampling time ST-III, the concentrations of almost all the elements increased compared to other sampling times, which was consistent with our previous research [3,40]. Although Si application may allocate more Na^+^ to the leaves via xylem, as was previously suggested by Bosnić et al. [102], and its foliar application has been connected to the lower Na plant intake in salinity stress [103], the results of this study were probably connected to the presence of Na in all the biostimulants treatment solutions used, in concentrations from 19.9 mg/L (Silitec) to 29.1 mg/L in Na selenate.

## 4. Materials and Methods

### 4.1. Field Experiment and Olive Leaf Sampling

A one-year field trial was conducted with the Leccino cultivar in an olive grove in full maturity, according to a randomized complete block design with four foliar fertilization treatments and four repetitions. The experiment was conducted on a total of sixteen uniform olive trees located on Kalkocambisol soil in Zadar County, Croatia. The soil’s chemical properties were analyzed following the methodology outlined by Pasković et al. [104] and were as follows: pH (H_2_O) = 7.2, pH (KCl) = 7.0, Organic matter = 2.6%, nitrogen(N) = 0.19%, plant available phosphorus (P) (mg/100 g) = 7, plant available K (mg/100 g) = 21. The olive trees were cultivated using the free-vase training system. Basic fertilization was implemented, including spring soil NPK fertilization and standard foliar fertilization practice with boron before flowering [3]. In addition, plant protection practices were carried out according to the principles of integrated pest management, which allowed for a sustainable and environmentally conscious approach to olive production [105].

In accordance with our preliminary trials and reports from other authors [22,53], four treatments were either water-treated control or foliar sprayed Se (50 mg/L of Se from sodium selenate (Na_2_SeO_4_) (Merck KGaA, Darmstadt, Germany), foliarly applied Si (1.1 g/L of Si from Silitec ^®^ (Kimitec Group, Almeria, Spain)), or their combination (Se+Si; the same concentration as for individual treatment). For suspension preparation, 8.5 mL/L of Silitec (which contains 10% K_2_O) or 119.63 mg/L of sodium selenate was diluted in water and applied, as noted before. For solution preparation, the surfactant Tensiofill (Agrofill, Lore, Italy) was used in all treatments, including the control, at a concentration of 0.1%. The plants were foliar-treated three times during the growing season (Table 7) in the morning using a backpack motorized sprayer with two nozzles (sprayer model: SOLO 433H, SOLO ^®^ Kleinmotoren GmbH, Stuttgart, Germany) with a volume of 20 L. Four whole trees per treatment were sprayed with approximately 10 L of treatment solution until runoff. During the application, air temperatures ranged from 18 to 25 °C for the first and second foliar spraying, and 16 and 22 °C for the third one. Simultaneously, the relative humidity for the area varied between 41 and 52% during July, 44 and 56% through August, and finally, 50 and 65% during September. All data on relative humidity were provided by the Croatian Meteorological and Hydrological Service (https://meteo.hr/—accessed on 9 December 2024).

Leaves from the current season were sampled from the central part of one-year-old olive shoots [22] at three different times (Table 7). Each average sample comprised 100 well-developed and healthy olive leaves, which were uniformly sampled considering all sides of the world. To remove impurities, we followed a procedure proposed by Poščić et al. [106]. Leaves were washed with 1% acetic acid, rinsed twice with distilled water, and dried in clean, marked paper bags in a dryer at 35 °C until reaching constant weight [2]. Dried leaves were ground and kept until further analysis, as previously described [3,40].

### 4.2. Chemicals

All solvents were of HPLC grade: both methanol and acetonitrile were purchased from Merck (Darmstadt, Germany). Phosphoric acid was obtained from Sigma-Aldrich (St. Louis, MO, USA). Chemical standards of phenolic compounds were of purity ≥98.0% (Extrasynthese, Genay, France). Deionized water was obtained by a Siemens UltraClear apparatus (Siemens AG, München, Germany). Argon used to form plasma for inductively coupled plasma mass spectrometric analysis (ICP-MS) was of purity 6.0 and, together with acetylene, was supplied by Messer (Messer Croatia Plin d.o.o., Zaprešić, Croatia).

### 4.3. High-Performance Liquid Chromatography (HPLC), Total Phenolic Content (TPC), and Total Antioxidant Capacity

Ground olive leaves (30 mg) were suspended in methanol (80% v/v, 1.5 mL) and extracted in an ultrasonic bath (Sonorex Digitec; Bandelin electronic, Berlin, Germany) for 15 min. The supernatants were separated after centrifugation for 5 min at 5000 rpm (Domel Centric 350; Železniki, Slovenia) and filtered through 0.45 μm cellulose filters. The HPLC analyses were conducted on a Shimadzu Nexera LC-40DX3 HPLC instrument (Shimadzu, Kyoto, Japan) with a SIL-40CX3 autosampler, a CTO-40C thermostatted column compartment, and an SPD-M40 photo diode array detector (DAD). The chromatographic separation was performed on a reverse-phase core–shell column C18 (2.1 mm × 150 mm, 2.7 µm particle size; Agilent Technologies, Palo Alto, CA, USA). The mobile phases consisted of 0.1% formic acid in water (A) and 0.1% formic acid in acetonitrile (B) with a gradient elution of 95%A–5%B for 0–2 min, 95%A–5%B for 2–20 min, 50%A–50%B for 20–21 min, 5%A–95%B for 21–23 min, 5%A–95%B for 23–24 min and then changed to 95%A–5%B for 24–30 min. The column temperature was set to 30 °C, and the flow rate of the mobile phase was 0.35 mL/min. The injection volume was 5 µL, and DAD chromatograms were recorded at 360 nm for luteolin-4-O-glucoside, luteolin-7-O-glucoside, apigenin-7-O-glucoside, apigenin, luteolin, and rutin, at 280 nm for oleuropein, oleuropein aglycone, oleacein, catechin, tyrosol, hydroxytyrosol, and verbascoside, and at 210 nm for oleanolic acid, respectively. Identification was performed by comparing the UV profile and retention time of the target compounds with those of chemical standards. Quantification was conducted using the external standard method. The calibration curves for individual phenolic compounds were obtained using five calibration levels made by appropriate dilutions of stock standard solutions, and calibration curves with R^2^ ≥ 0.999 were accepted for concentration calculation [107].

Total phenolic content (TPC) was assessed using the Folin–Ciocalteu assay [108]. In short, 20 µL of an extract was mixed with 140 µL of 0.2 M Folin–Ciocalteu reagent. After 1 min, 140 µL of 6% sodium carbonate was added, and the mixture was left at 25 °C for 60 min. Absorbance was then measured at 750 nm using a microplate reader (Tecan Infinite 200 Pro M Nano+, Männedorf, Switzerland). Results were quantified using a calibration curve with serial dilutions of gallic acid (GA; 12.5, 25, 50, 75, 100, 150, 250 mg/L; coefficient of determination, R^2^ = 0.9999) and expressed as mg GA equivalents/g dry weight (DW).

Total antioxidant capacity was evaluated by the 2,2-diphenyl-1-picrylhydrazyl radical scavenging activity (DPPH) assay [109] and the ferric-reducing ability of the plasma (FRAP) assay [110]. For the DPPH assay, 100 µL of the extract was mixed with 200 µL of freshly prepared 0.02 M DPPH in a 96-well plate. Absorbance was measured at 517 nm after 30 min of reaction time at 25 °C. The FRAP assay involved mixing 100 µL of the sample with 200 µL of FRAP reagent in the same plate, and absorbance was read at 593 nm after 10 min of reaction time at 25 °C. Measurements were performed using the microplate reader Infinite 200 Pro M Nano+ ().

### 4.4. Inductively Coupled Plasma Mass Spectrometry (ICP-MS)

Approximately 0.2 g of dried and finely grounded leaves from each sample was digested using an Anton Paar Multiwave 3000 microwave system (Anton Paar GmbH, Graz, Austria) equipped with pressurized vessels. The samples were digested with 5 mL of 65% nitric acid per sample (Suprapur, Merck, Germany) over a 20 min operation cycle at 200 °C. After cooling, the digested samples were transferred to 25 mL volumetric flasks, and ultrapure water was added to the mark.

The concentrations of Cu, Fe, I, Se, Si, and Zn were determined by an ICP-MS instrument NexION 300X equipped with an S10 autosampler (PerkinElmer Instruments, Waltham, MA, USA). Multielement tuning solution (NexION Setup Solution, PerkinElmer Instruments) was used for performance check and tuning, covering a wide range of the masses of the elements. The concentrations of elements were determined using the external standard method with matrix-matched calibration. Multielement standard solution (PerkinElmer Instruments) was used to prepare calibration curves. The concentrations of Ca, K, Mg, and Na were determined by an atomic absorption spectrometer AAS800 (PerkinElmer Instruments) using the flame atomization technique. Single-element standard solutions (Inorganic Ventures, Christiansburg, VA, USA) were used to calibrate the instrument. Analytical blanks were prepared and run in the same way as the samples. The calibration curves with R^2^ > 0.999 were accepted for concentration calculation.

### 4.5. Liquid Chromatography–Mass Spectrometry (LC-MS)

Following the extraction of samples for phenolic content analyses, an aliquot was separated and dried in a lyophilizer and then reconstructed by adding H_2_O and 0.1% formic acid. The primary metabolite profile was determined using an LC-MS/MS system, which included an autosampler (Shimadzu Nexera SIL-40CX3, Kyoto, Japan), two solvent delivery units (Shimadzu Nexera LC-40DX3, Kyoto, Japan), a thermostatic column compartment (Shimadzu Nexera CTO-40C, Kyoto, Japan), and a triple-quadrupole mass spectrometer (Shimadzu LCMS8045, Kyoto, Japan). Separation was carried out on a Discovery^®^ HS F5-3 column (2.1 mm × 150 mm, 3 µm core–shell, Sigma-Aldrich, St. Louis, MO, USA) at 37 °C. A 1 µL sample was injected, and separation was achieved using a linear gradient of mobile phase A (water with 0.1% formic acid) and mobile phase B (acetonitrile with 0.1% formic acid) at a flow rate of 0.25 mL/min. The gradient program was as follows: 0–2 min: 100% A; 2–5 min: 100% A to 75% A; 5–11 min: 75% A to 65% A; 11–15 min: 65% A to 5% A; 15–20 min: 5% A; 20–20.1 min: 5% A to 100% A; and 20.1–25 min: 100% A. The identification and quantification of targeted compounds were performed by comparing retention times, characteristic precursor/product ion pairs, and peak areas to reference standards.

### 4.6. Statistical Analysis

Raw data were evaluated with repeated measurements ANOVA with sampling times (ST) as a repeated factor. When significant, Tukey’s post hoc test was applied and significant differences between means (*n* = 4) were considered at *p* < 0.05. Pearson’s correlations matrix was generated between selected elements, IAA, phenolic compounds, and other metabolites. These statistical analyses were performed using the TIBCO Statistica 14.1.0.8 software (TIBCO StatSoft^®^, Palo Alto, CA, USA) and SAS software 14.2 (SAS Institute Inc., 2016, Cary, NC, USA). In addition, multivariate statistical analysis was conducted using partial least squares–discriminant analysis (PLS–DA). PLS–DA is a supervised multivariate statistical method that provides information on the most meaningful variables (e.g., phenols, minerals, amino acids, or other selected metabolites) in the form of variable importance in projection (VIP) scores. It minimizes the variance within and maximizes the variance between different categories, such as varieties or sampling times. This analysis was performed in MetaboAnalyst v. 6.0 [111].

## 5. Conclusions

This study showed that foliar application of selenium, silicon, and their combination can enhance the synthesis and increase the concentrations of phenolic compounds in olive leaves, including important antioxidants such as oleuropein and other secoiridoids, as well as flavonoids. The effects varied depending on the treatment and the compound, but in most cases resulted in leaves with significantly increased biological potential, suitable for use as an enhanced raw material for various purposes. Some of the mentioned effects were shown to significantly depend on the sampling times and were, in most cases, the strongest after three consecutive treatments, implying a cumulative effect of repeated selenium and silicon foliar application. It was also observed that the combined supplementation of selenium and silicon results in specific changes, suggesting an interplay between the effects of these elements during plant development under such circumstances. Certain inter-relationships between the applied treatments, concentration of phenolic compounds, and their aromatic amino acid and other precursors were also noted. The majority of the investigated phenols correlated positively with aromatic amino acids, which was assumed to be mostly related to the joint increase in their concentrations at the last sampling time during olive harvest. The response of verbascoside to the applied treatments appeared to be closely linked to corresponding changes in its amino acid precursors. On the other hand, it was challenging to clearly link the changes in secoiridoid levels with those observed in aromatic acids and other related compounds, likely due to the complex dual nature of secoiridoid biosynthesis.

In addition to advancing knowledge on the possibilities for olive leaf biofortification, this study provided new insights that could help clarify the biochemical responses of phenolic metabolism in olives to selenium and silicon foliar fertilization. Future studies should focus on elucidating the effects of such treatments on other olive plant tissues and products, such as fruit and oil, but also on the potential selenium toxicity in olive leaf infusions or similar products for human or animal consumption due to the possible presence of sodium selenate.

## Figures and Tables

**Table 1:** Effect of different foliar treatments, sampling times, and their interactions on antioxidant capacity and total phenols and the sum of HPLC determined phenols in olive (*Olea europaea* L., Leccino cv.) leaves treated thrice with a solution of selenium (Se), silicon (Si), or their combination (Se+Si).

	Antioxidant Capacity (µmol TE */g DW)		Total Phenols mg/100 g DW	Sum of HPLC Phenols
	FRAP	DPPH		mg/100 g DW
**Treatments (T)**				
Control	1687.77 ± 9.60	207.41 ± 7.35	2088.75 ± 162.16	4887.61 ± 418.47 ^b^
Se	168.47 ± 6.39	205.60 ± 4.55	2207.33 ± 147.29	5525.47 ± 395.81 ^ab^
Se+Si	167.26 ± 10.33	202.52 ± 8.57	2472.08 ± 188.95	6362.5 ± 498.5 ^a^
Si	170.17 ± 8.16	213.15 ± 7.38	2391.92 ± 176.8	6111.84 ± 334.67 ^a^
*p*-value	0.992	0.332	0.108	**0.004**
**Sampling times (ST)**				
August (ST-I)	158.16 ± 5.73 ^b^	208.50 ± 3.97 ^a^	1989.06 ± 107.11 ^b^	5182.73 ± 164.44 ^b^
September (ST-II)	150.12 ± 4.27 ^b^	191.49 ± 6.07 ^b^	2020.00 ± 82.57 ^b^	4554.03 ± 239.07 ^c^
October (ST-III)	196.97 ± 5.83 ^a^	221.51 ± 5.63 ^a^	2861.00 ± 129.78 ^a^	7428.8 ± 238.47 ^a^
*p*-value	**<0.001**	**<0.001**	**<0.001**	**<0.001**
**Treatments × Sampling times**				
Control × ST-I	162.78 ± 13.16	224.06 ± 4.86 ^ab^	1780.75 ± 408.54	4543.33 ± 252.92 ^fg^
Control × ST-II	148.11 ± 15.09	177.57 ± 4.68 ^cd^	2128.75 ± 70.99	3500.34 ± 110.99 ^g^
Control × ST-III	192.42 ± 16.51	220.60 ± 10.10 ^abc^	2356.75 ± 247.40	6619.16 ± 411.77 ^bc^
Se × ST-I	158.93 ± 5.81	203.64 ± 6.57 ^abcd^	1933.00 ± 102.20	5224.24 ± 248.34 ^def^
Se × ST-II	152.02 ± 2.99	210.08 ± 6.08 ^abcd^	1934.75 ± 126.40	4190.21 ± 164.91 ^fg^
Se × ST-III	194.45 ± 7.84	203.08 ± 11.74 ^bc^	2754.25 ± 250.32	7161.96 ± 342.28 ^bc^
(Se+Si) × ST-I	152.38 ± 11.95	211.02 ± 9.24 ^abcd^	2113.75 ± 77.70	5941.51 ± 236.04 ^cd^
(Se+Si) × ST-II	141.26 ± 7.46	172.8 ± 9.82 ^d^	2047.75 ± 193.33	4631.91 ± 244.65 ^efg^
(Se+Si) × ST-III	208.13 ± 10.98	223.72 ± 12.53 ^ab^	3254.75 ± 205.66	8514.06 ± 132.09 ^a^
Si × ST-I	158.55 ± 16.97	195.29 ± 3.68 ^bcd^	2128.75 ± 137.41	5021.86 ± 148.41 ^def^
Si × ST-II	159.09 ± 4.17	205.5 ± 14.86 ^abcd^	1968.75 ± 265.43	5893.64 ± 171.3 ^cde^
Si × ST-III	192.87 ± 12.98	238.65 ± 4.79 ^a^	3078.25 ± 121.34	7420.01 ± 445.35 ^ab^
*p*-value	0.810	**0.001**	**0.440**	**<0.001**

Results are expressed as means ± standard errors (*n* = 4). Different letters for each variable indicate significant differences between mean values for treatment, sampling time, and their interaction (*p* ≤ 0.05) using RM ANOVA and Tukey’s test, the absence of letters represents an insignificant result. * TE (Trolox equivalent).

**Table 7:** Temporal dynamics of foliar treatments of olive (*Olea europaea* L. cv. Leccino) with Se and Si and respective leaves sampling.

Date	Treatments and Sampling
July	First treatment (50 days after anthesis)
August	First sampling (ST-I; 30 days after first treatment)
August	Second treatment (80 days after anthesis)
September	Second sampling (ST-II 30 days after second treatment)
September	Third treatment (110 days after anthesis)
October	Third sampling (ST-III; 30 days after the third treatment, i.e., at the technological maturity of the fruit coinciding 140 days after anthesis)

## Data Availability

The data supporting our findings and analyzes are contained in the article itself. Readers can access these data by referring to the article.
